# Deep Sequencing Whole Transcriptome Exploration of the σ^E^ Regulon in *Neisseria meningitidis*

**DOI:** 10.1371/journal.pone.0029002

**Published:** 2011-12-15

**Authors:** Robert Antonius Gerhardus Huis in 't Veld, Antonius Marcellinus Willemsen, Antonius Hubertus Cornelis van Kampen, Edward John Bradley, Frank Baas, Yvonne Pannekoek, Arie van der Ende

**Affiliations:** 1 Department of Medical Microbiology, Center of Infection and Immunity Amsterdam (CINIMA), Academic Medical Center, Amsterdam, The Netherlands; 2 Bioinformatics Laboratory, Department of Clinical Epidemiology, Biostatistics and Bioinformatics, Academic Medical Center, Amsterdam, The Netherlands; 3 Biosystems Data Analysis, Swammerdam Institute for Life Science, University of Amsterdam, Amsterdam, The Netherlands; 4 Department of Genome Analysis, Academic Medical Center, Amsterdam, The Netherlands; 5 Reference Laboratory for Bacterial Meningitis, Academic Medical Center, Amsterdam, The Netherlands; National Taiwan University Hospital, Taiwan

## Abstract

Bacteria live in an ever-changing environment and must alter protein expression promptly to adapt to these changes and survive. Specific response genes that are regulated by a subset of alternative σ^70^-like transcription factors have evolved in order to respond to this changing environment. Recently, we have described the existence of a σ^E^ regulon including the anti-σ-factor MseR in the obligate human bacterial pathogen *Neisseria meningitidis*. To unravel the complete σ^E^ regulon in *N. meningitidis*, we sequenced total RNA transcriptional content of wild type meningococci and compared it with that of *mseR* mutant cells (Δ*mseR*) in which σ^E^ is highly expressed. Eleven coding genes and one non-coding gene were found to be differentially expressed between H44/76 wildtype and H44/76Δ*mseR* cells. Five of the 6 genes of the σ^E^ operon, *msrA/msrB*, and the gene encoding a pepSY-associated TM helix family protein showed enhanced transcription, whilst *aniA* encoding a nitrite reductase and *nspA* encoding the vaccine candidate Neisserial surface protein A showed decreased transcription. Analysis of differential expression in IGRs showed enhanced transcription of a non-coding RNA molecule, identifying a σ^E^ dependent small non-coding RNA. Together this constitutes the first complete exploration of an alternative σ-factor regulon in *N. meningitidis*. The results direct to a relatively small regulon indicative for a strictly defined response consistent with a relatively stable niche, the human throat, where *N. meningitidis* resides.

## Introduction

Bacteria live in an ever-changing environment and must alter protein expression promptly to adapt to these changes and survive. Specific response genes that are regulated by a subset of the alternative σ^70^-like sigma factors have evolved in order to respond to this changing environment [1]. In reaction to specific external stimuli, these sigma factors recruit RNA polymerases to the appropriate response genes. The name extracytoplasmic function (ECF) sigma factors or σ^E^ factors refer to the fact that most of the genes under control of these σ factors encode proteins residing in the outer membrane or periplasmic space [2].

Recently, using a proteomic approach, we have described the existence of a σ^E^ regulon in the obligate human bacterial pathogen *Neisseria meningitidis* [3]. The σ^E^ operon so far identified encompasses an operon consisting of 6 genes (NMB2140-NMB2145) among which the gene encoding σ^E^ itself (NMB2144). In addition *msrA/msrB* (NMB0044) was also found to be subjected to regulation by σ^E^, it encodes for methionine sulfoxide reductase, an enzyme repairing proteins exposed to reactive oxygen species (ROS) [4], [5]. Furthermore, the anti-σ^E^ factor MseR (for Meningococcal SigmaE Regulator; NMB2145) was identified. Deletion of *mseR* results in overexpression of the other 5 genes in the σ^E^ operon, thereby increasing the expression of *σ^E^* itself as well as *msrA/msrB* [3]. The proteomic exploration of the σ^E^ regulon of meningococci and a microarray gene expression analysis for the closely related human pathogen *Neisseria gonorrhoeae* performed by others have revealed a surprisingly small regulon for the σ^E^ factor in *Neisseria* [6]. However, both methods have several inherent and technical limitations. Identification of proteins is limited to those that can be isolated efficiently, are within a certain size range resolvable with SDS-PAGE, and have to be sufficiently expressed to be both visible and able to be confidently identified using mass spectrometry. DNA microarrays are only able to detect products that can sufficiently hybridize with probes that have been created based on available genomic data, usually limited to *in silico* predicted open reading frames (ORFs) of protein coding genes in their sense orientation. Moreover, reliability and reproducibility remains a concern [7]–[9]. Both approaches are furthermore unsuitable to detect an important class of RNA regulators called small RNAs (sRNA) [10], [11]. Previous investigations in *Salmonella enterica* and *Escherichia coli* have shown several sRNAs to be regulated by their respective σ^E^ orthologues, making it essential to be able to detect these transcripts in any complete exploration of σ-factor regulation [12], [13]. RNA-seq using next generation sequencing has the potential to overcome all mentioned limitations by offering an approach to identify all RNA species expressed within a cell without pre-selection [14], [15]. Therefore, in order to unravel the complete σ^E^ regulon in *N. meningitidis*, we sequenced total RNA transcriptional content of wild type meningococci and compared it with that of *mseR* knock-out cells in which σ^E^ is highly expressed.

## Materials and Methods

### Bacterial strains and growth conditions

*N. meningitidis* strain H44/76, B:P1.7,16:F3-3: ST-32 (cc32), is closely related to serogroup B strain MC58, belonging to the same clonal complex [16]–[20]. *N. meningitidis* H44/76 in which *mseR* was replaced with the erythromycin resistance cassette *ermC* (H44/76Δ*mseR*) was described previously [3]. Meningococci were grown on GC plates (Difco), supplemented with 1% (vol/vol) Vitox (Oxoid) at 37°C in a humidified atmosphere of 5% CO_2_. Broth cultures were incubated in GC medium supplemented with 1% (vol/vol) Vitox on a gyratory shaker (180 rpm) at 37°C. Where appropriate, plates were supplemented with erythromycin (5 µg/mL). Growth was monitored by measuring optical density of cultures at 530 nm (OD_530_, Pharmacia Biotech Ultraspec 2000) at regular intervals. For RNA isolation, used in both RNA-seq and qRT-PCR experiments, cells were grown to exponential growth phase (OD_530_ = 0.5–0.6).

### Total RNA preparation

Immediately after sample removal, RNA was stabilized by adding 1/10^th^ volume of stop solution (95% ethanol/5% phenol pH 4.3) and samples were further processed after rapid cooling on ice [21]. Isolation of total RNA was performed using an acidic hot phenol method adapted from [22]. RNA molecules smaller than 200 nucleotides (nts), including 5S and tRNAs, were retained using classic acidic phenol/chloroform extractions for sample clean-up [23]. The majority of 16S and 23S rRNA molecules were selectively removed using the MICROBExpress kit (Life Technologies, 2008). RNA quality was assessed by resolving samples on 1% agarose gels and measuring RNA Integrity Number using a 2100 Bioanalyzer (Agilent Technologies) [24]. For RNA-seq experiments RNA with a RIN value ≥9.0 was used. Quantification was assessed by UV spectroscopy (Nanodrop 1000, Thermoscientific).

### SOLiD sequencing

Transcriptome sequencing was performed according to the manufacturer's protocol using 1 µg RNA isolated from H44/76 wild type (H44/76 wt) or H44/76Δ*mseR* cells (SOLiD Whole Transcriptome Analysis Kit, Life Technologies, 2009 Rev. F). Adaptor mix A was used for RNA-adaptor ligation, ensuring strand specificity. In short, this is achieved by ligating the fragmented RNA molecules using double stranded adapters with single stranded random hexamers overhangs either on the 5′ or the 3′ end. Subsequent sequencing is performed using primers that anneal to only one of these two adapters. Bead preparation was performed according to the SOLiD v3 Plus system's instructions (Life Technologies, 2009). The capacity of a single full slide was used, using barcodes to individually tag two H44/76 wt and two H44/76Δ*mseR* technical replicates, consisting of separately constructed cDNA libraries.

### qRT-PCR

After DNase treatment (Roche, 2008) 2.5 µg RNA was used as input for reverse transcription, performed as per ThermoScript kit instructions (Invitrogen, 2010) with a single adaptation: all procedures were performed *in duplo*, substituting RT with nuclease-free water in 1 reaction to serve as no-RT controls. The resulting RT+ and RT- cDNA samples were serially diluted and PCR amplification of the constitutively expressed outer membrane protein *rmpM* was used to titrate relative total cDNA concentrations of the H44/76 wt and H44/76Δ*mseR* samples. The cDNA samples were adjusted accordingly for use in subsequent real-time PCRs (Lightcycler, Roche) (see Table S1 for primers used). Crossing point (Cp) values were calculated manually for all genes using the Fit Points Method in the LightCycler Software package (Roche, version 4.05) [25], [26]. All experiments were carried out *in triplo*. To normalize target gene Cp values, mean Cp values of the internal control *rmpM* of both H44/76 wt and H44/76Δ*mseR* were deducted from the Cp value of the respective target genes. Unpaired two-sample t-tests were performed for assessing the statistical significance of the difference between the means of these normalized Cp values. This was followed by a one-tailed test to derive *p*-values with 0.05 chosen as the threshold for statistical significance.

### Data analysis

The SOLiD Accuracy Enhancer Tool (SAET) was used to correct miscalled bases and subsequently the P2 sequencing adapter was trimmed using a tool provided by Nicholas Socci (www.pyrodigm.com). The corrected and trimmed reads were mapped to the recently published H44/76 Whole Genome Shotgun (WGS) sequence (GenBank accession AEQZ00000000) using BWA [19], [27]. This mapping was performed in two steps. First the reads were mapped with default BWA settings (version 0.5.7 r1310). Subsequently the unmapped but high quality reads are mapped in a second step with less stringent settings allowing for 2 mismatches in the first 25 nts and 4 mismatches for the total read (up to 50 bp). These mismatches were allowed to compensate for limited genome sequence errors, i.e., the reference H44/76 WGS genome sequence was obtained by 454 pyrosequencing prone to inaccurate sequencing of long homopolymer stretches, and to allow possible mapping of post-transcriptionally altered RNA molecules [19]. The results of these mappings were merged for further analysis using SamTools [28]. For every ORF and intergenic region (IGR) we counted the number of aligned reads using the Rsamtools package [29], [30]. Reads that overlapped with two ORFs and/or IGRs both contributed to the read count of these two ORFs/IGRs. Reads that could not be unambiguously mapped to a single location were ignored in subsequent analysis. This concerned an estimated 2% of the genome involving repetitive sequences and duplicated genes like transposase elements (73 annotated), phage related proteins (13 annotated), iron regulated proteins FrpA/B/C/D (includes several pseudogenes), the iron uptake system TpbA/B (duplicated), maf type adhesins (duplicated), Type IV pili, and rRNA genes (one copy annotated) as H44/76 does not contain large duplicated regions [19]. Library normalization and differential expression analysis for all IGRs larger than 29 nts and all ORFs on both strands was performed using DEseq [31]. For ORFs this resulted in the separate analysis of both sense and anti-sense transcription. DEseq settings were optimized for the most conservative analysis of technical replicates as described by Anders *et al.* [31]. The library sizes of the different samples were adjusted based on the total number of reads per library, without the use of size factor estimation. The *p*-values resulting from the pairwise comparison between H44/76 wt and H44/76Δ*mseR* were adjusted by the Benjamini-Hochberg procedure to control the false discovery rate (FDR) [32]. Visual inspection of mapped reads was facilitated with the Integrative Genomics Viewer (16).

*In silico* prediction of transcriptional start sites (TSSs) was performed using the Neural Network Promoter Prediction program (prokaryote organism setting), ρ-independent terminators were identified manually using Mfold or adapted from those predicted by TransTerm HP in *N. meningitidis* MC58 [33]–[35]. All reported nt positions are according to the published H44/76 whole genome shotgun sequence [19]. Potential mRNA targets (in the reference strain MC58) of sRNAs were predicted *in silico* using TargetRNA, its default settings adjusted only to allow for G:U pairs in seed [36].

## Results

### Output SOLiD

The total number of reads varied from 50×10^6^ to 92×10^6^ (Table 1). The proportion of mapped reads ranged from 50% to 68%, with the second less stringent mapping accounting for 4–6% of all mapped reads. Thirty percent to 47% of the reads mapped to tRNA or rRNA genes. In one technical replicate, H44/76Δ*mseR* replicate A, the proportion of reads mapped to tRNA/rRNA genes was lower compared to the other 3, while the proportion of unmapped reads was higher. This might be attributable to slightly different gel size fractioning; however this has no observable effect on the total reads mapped to the rest of the genome. Raw read mapping analysis (before library normalization) showed at least 1 read could be mapped to 2593/2598 (100%) ORFs and 1635/2599 (63%) IGRs, with 43% of the positive and 48% of the negative strand covered by 10 or more reads.

**Table 1:** Mapping statistics of SOLiD output.

	Number of reads ×10^6^ in cells of			
	H44/76 wt		H44/76Δ*mseR*	
Reads	replicate A (%)	replicate B (%)	replicate A (%)	replicate B (%)
**Mapped to genome**				
Excluding tRNA/rRNA	20 (24%)	12 (21%)	18 (20%)	12 (24%)
tRNA/rRNA genes only	37 (44%)	27 (47%)	28 (30%)	22 (44%)
**Unmapped**	27 (32%)	19 (33%)	46 (50%)	16 (32%)
**Total**	84 (100%)	58 (100%)	92 (100%)	50 (100%)

Two technical replicates were performed for both H44/76 wt and H44/76Δ*mseR*, resulting in a total of 280×10^6^ reads.

### Differentially expressed genes

Eleven coding genes and one non-coding gene were found to be differentially expressed between H44/76 wt and H44/76Δ*mseR* cells with a corrected *p*-value≤0.05 (Table 2).

**Table 2:** Differentially expressed loci, ranked by locus number, between *N. meningitidis* H44/76 wt and H44/76Δ*mseR*.

		reads/locusα				
Locusβ	Geneγ	wt	Δ*mseR*	Fold change	*p*-valueδ	Descriptionε
NMH_0518		1998	5686	2.8	0.010	pepSY-associated TM helix family protein
NMH_1358		3740	1403	0.4	0.021	outer membrane OpcA family protein
NMH_2149		5216	187292	35.9	<0.0001	doxX family protein, σ^E^ operon
NMH_02153		5042	60516	12.0	<0.0001	Hypothetical protein in σ^E^ operon
NMH_2154		4807	63164	13.1	<0.0001	Hypothetical protein in σ^E^ operon
NMH_2155		3071	40218	13.1	<0.0001	Hypothetical protein in σ^E^ operon
NMH_2156	*σ^E^*	1587	9997	6.3	<0.0001	RNA polymerase σ^E^, σ^E^ operon
NMH_2157	*mseR*	331	-ζ	n/a	n/a	Meningococcal σ^E^ Regulator, σ^E^ operon
NMH_2253	*nirK*	638	117	0,2	<0.0001	*aniA* (MC58)
NMH_2270	*opA50*	1098	418	0,4	0.027	*nspA* (MC58)
NMH_2477	*msrA*	1726	138968	80.5	<0.0001	methionine sulfoxide reductase *msrA/msrB*
σ^E^ sRNA		7	114	16.3	<0.0001	σ^E^sRNA

α Reads/locus after library normalization by DEseq.

β Locus number as annotated in H44/76 WGS (AEQZ00000000).

γ Gene name as annotated in H44/76 WGS (AEQZ00000000).

δ All loci with Benjamini-Hochberg adjusted p-value≤0.05.

ε Where available *N. meningitidis* strain MC58 orthologues are noted.

ζ *mseR* artificially deleted.

**Figure 1:**
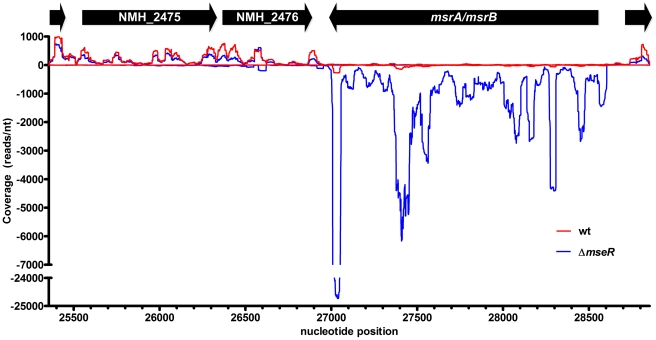
Read coverage visualization of the upregulation of *msrA/msrB* in H44/76Δ*mseR* (blue) versus H44/76 wt (red). Transcription on the + strand is visualized on the positive x-axis, - strand transcription on the negative x-axis. Nucleotide position refers to contig9 of H44/76 WGS (AEQZ01000046.1). The relatively high peak of the last 50 nucleotides of *msrA/msrB* is present in both H44/76 wt and H44/76Δ*mseR* in both replicates. Expression of *msrA/msrB* through its ρ-independent terminator can be seen (see text for details), explaining the upregulation of anti-sense transcription of NMH_2475 and NMH_2476 shown in table 3.

**Figure 2:**
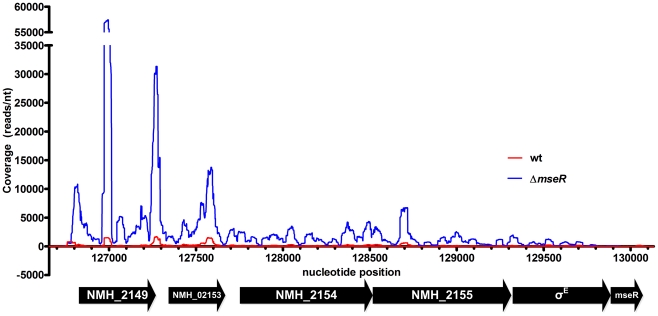
Read coverage visualization of the upregulation of the σ^E^ operon in H44/76Δ*mseR* (blue) versus H44/76 wt (red). Transcription on the + strand is visualized on the positive x-axis, - strand transcription on the negative x-axis. Nucleotide position refers to contig6 of H44/76 WGS (AEQZ01000043.2). The largest absolute transcriptional differences can be found in the first 2 genes of the σ^E^ operon, NMH_2149 and NMH_02153.

In contrast to H44/76 wt cells, in H44/76Δ*mseR* cells no reads were detected that mapped to the *mseR* locus of the H44/76 reference sequence (Table 2) [19]. In addition, RNA from H44/76 wt cells did not contain reads that mapped to *ermC*, while H44/76Δ*mseR* cells in which *mseR* was replaced by *ermC* yielded reads that mapped to this locus (data not shown). Therefore, differential transcription of artificially introduced and removed genes was confidently identified. Previously, using RT-PCR and proteomics, we identified the σ^E^ operon and *msrA/msrB* to be upregulated in H44/76Δ*mseR* cells [3]. In the current RNA-seq analysis the expression of *msrA/msrB* (Figure 1) and the genes comprising the σ^E^ operon (Figure 2) was 81-fold and between 6-fold and 36-fold increased, respectively (Table 2). Of interest, four differentially expressed genes were identified which were hitherto not associated with σ^E^. Of these, one was upregulated: NMH_0518 (analogous to NMB1721; encoding a hypothetical protein of the PepSY-associated TM helix family of proteins). Three genes, NMH_2270 (analogous to NMB0663; *nspA*), *nirK* (analogous to NMB1623; *aniA*), and NMH_1358 (analogous to NMB1053; *opcA* outer membrane protein), appeared to be downregulated (Table 2). However, the promoter of the latter gene contains a homopolymeric cytidine tract located at the -35 region which can be extended or shortened during DNA replication by a mechanism called slipped strand DNA replication [37], [38]. As a result, *opcA* displays variable expression [37]. Indeed, classic Sanger re-sequencing of this location revealed the promoter of H44/76 wt *opcA* contained a tract of 14 cytosines, while that of H44/76Δ*mseR* contained 15, the latter causing severe reduced transcriptional efficiency [37]. Therefore, we considered differential expression of NMH_1358 most likely to be the result of unrelated phase-variation independent of σ^E^ expression. A complete list of differentially expressed loci with uncorrected *p*-values≤0.01 can be found in Table S2. Among the 30 loci with differential sense expression, four clusters of genes and their associated IGRs, either physically arranged in an operon or with a functional relationship, were recognized; cluster 1 comprising three tRNA-Leu genes (NMH_1084, NMH_1085, and NMH_2191); cluster 2 consisting of genes encoding a restriction modification system (NMH_1821-NMH_1823), cluster 3 encoding a leukotoxin type I secretion system (NMH_1851, NMH_0534-NMH_0535) and cluster 4 encoding genes involved in a thiamin biosynthesis operon (NMH_2073, NMH_2074, NMH_2076, *thiG*). In these clusters the difference in expression between H44/76 wt and H44/76Δ*mseR* cells varies between 2-fold and 3.4-fold. Statistical analysis of the total expression levels of these clusters (i.e., using the total read count of all genes in a cluster) did not result in additional significant differential expression considering the corrected *p*-value. Visual inspection of pairs of 100 randomly picked non-differentially expressed genes using the Integrative Genomics Viewer software revealed consistently highly similar patterns of read distribution between H44/76 wt and H44/76Δ*mseR* normalized libraries, indicating that variation between strains is limited.

### Differential transcription in intergenic regions

**Figure 3:**
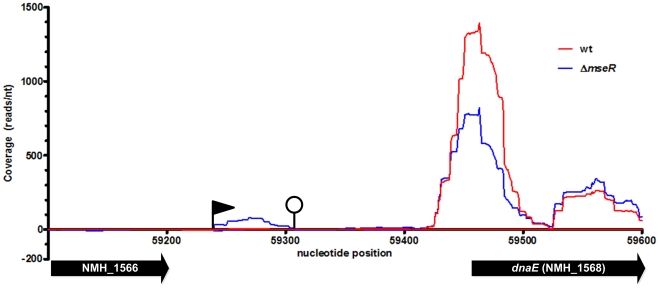
Read coverage visualization of the upregulation of the σ^E^sRNA in H44/76Δ*mseR* (blue) versus H44/76 wt (red). Transcription on the + strand is visualized on the positive x-axis, - strand transcription on the negative x-axis. Nucleotide position refers to contig4 of H44/76 WGS (AEQZ01000037.1). The black flag indicates a predicted TSS, the closed circle indicates a predicted ρ-independent terminator. The ORF of hypothetical protein NMH_1566 shows no expression. *dnaE* (NMH_1568), truncated in this picture with only 150 of 3435 nts shown, is not differentially expressed.

**Figure 4:**
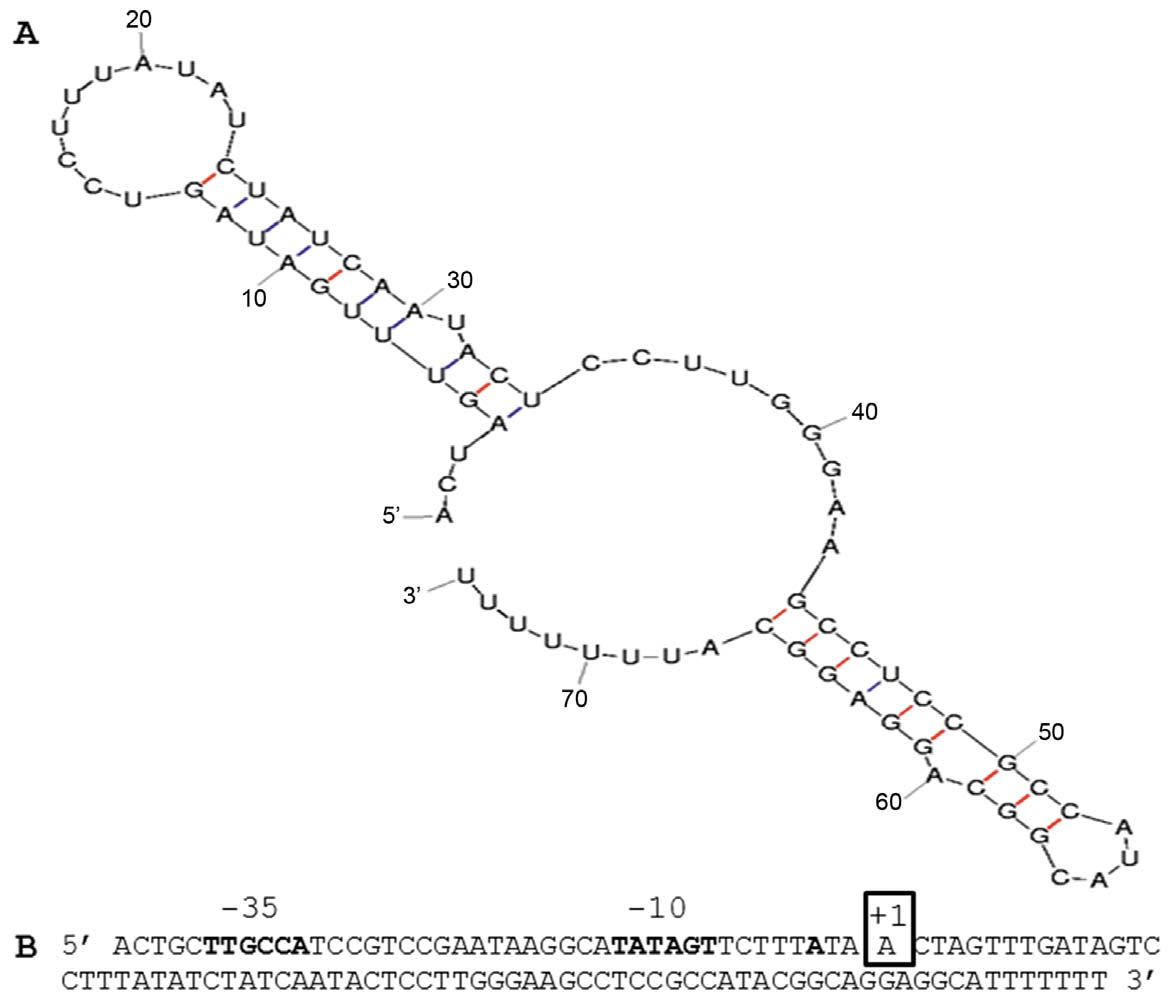
Sequence and predicted secondary structure of the σ^E^sRNA. (A) Mfold prediction of the secondary structure of the σ^E^sRNA. (B) Sequence of the σ^E^sRNA gene including promoter. The boldfaced -35 and -10 regions and transcriptional start site are predicted *in silico*. The boxed +1 nucleotide is the experimentally validated transcriptional start site of the *in vivo* present transcripts.

IGRs may contain non-coding RNAs involved in the riboregulation of target mRNAs [10], [11]. Of all IGRs of H44/76, only the IGR between NMH_1566 and NMH_1568 (analogous to NMB1826 and *dnaE* in MC58) showed differential expression with 16 times higher expression in H44/76Δ*mseR* versus H44/76 wt cells (Table 1). A gene encoding a sRNA of 74 nts can be identified in this IGR (Figure 3). Its TSS deduced from read mapping in RNA-seq is at contig4-nt position 59241 (AEQZ01000037.1), which is 11 nts downstream of an *in silico* predicted -10 region of a putative promoter and 3 nts downstream of the predicted TSS (Figure 4). The transcript ends within the T-tract of a ρ-independent terminator, with the last reads ending at contig4 nt position 59314 (Figure 4). The gene encoding this sRNA was found to be conserved in all currently sequenced complete and WGS genomes of *N. meningitidis, N. gonorrhoeae* and *N. lactamica* strains (query coverage 100%, identity ≥94%) and in several but not all commensal *Neisseria* species. Therefore, putative interaction between this sRNA and the 5′ UTR of target mRNAs was assessed using TargetRNA in the related strain MC58 [36]. This way, NMB0205 (*fur*), NMB0792 (*nadC*), NMB0810 (encoding a putative TetR family transcriptional regulator) and NMB1224, NMB1914, NMB2014, and NMB2110, all encoding hypothetical proteins, were identified as putative targets of this novel sRNA.

Differential expression between H44/76 wt and H44/76Δ*mseR* of NMH_0518, NMH_2270, *nirK*, *msrA* and NMH_2149 (representing the σ^E^ operon) was also assessed by qRT-PCRs on reverse transcribed RNA. The internal control *rmpM* showed very similar Cp values for both cDNA samples (11.7±1.4 vs 11.9±1.5; *p* = 0.4573), consistent with RNA-seq results showing less than 6% difference between total normalized reads mapped to *rmpM* (data not shown). Four genes showed similar direction of differential expression (three significant), confirming the RNA-seq data, although fold differences show varying order of magnitude in comparison (Table S3) [39]. Detection of transcription of NMH_2270 and the σ^E^ sRNA was insufficient to yield reliable results.

### Antisense transcription

*Cis*-antisense transcripts of genes have been implicated to interact with its mirror image mRNA molecule, regulating transcription, translation or degradation [15], [40]. We investigated differential expression by considering the number of reads per locus in the anti-sense direction of all ORFs and detected 6 genes to have differentially expressed anti-sense transcripts (Table 3). In H44/76 wt cells 771 reads were mapped to the anti-sense strand of *msrA/msrB*, while in H44/76Δ*mseR* cells this number was 6.4 fold reduced. In H44/76Δ*mseR* cells, antisense transcription of both NMH_2475 and NMH_2476 was enhanced 27 and 120 fold, respectively, possibly due to read-through from the strongly transcribed *msrA/msrB* locus (Figure 1). In addition, an 8.3 fold reduction in antisense transcription of NMH_2156 (part of the σ^E^ operon) was observed in H44/76Δ*mseR* cells. Lastly, antisense transcription of *nqrE* (NMB0565, Na^+^-translocating NADH-quinone oxidoreducase subunit E) and *σ^E^* itself was 4.0 and 5.4 fold increased, respectively.

**Table 3:** Differentially expressed loci in anti-sense direction, ranked by locus number, between *N. meningitidis* H44/76 wild type and *N. meningitidis* H44/76Δ*mseR*.

		reads/locusα				
Locusβ	Geneγ	wt	Δ*mseR*	Fold change	*p*-valueδ	Descriptionε
NMH_0763	*nqrE*	30	121	4.0	0.020	NADH: ubiquinone oxidoreductase
NMH_2154		157	19	0.1	<0.001	Hypothetical protein in σ^E^ operon
NMH_2156	*σ^E^*	26	141	5.4	0.001	RNA polymerase σ^E^, σ^E^ operon
NMH_2475		17	461	27.1	<0.001	mechanosensitive ion channel family protein
NMH_2476		6	725	120.8	<0.0001	Competence/damage-inducible CinA C-terminal
NMH_2477	*msrA/msrB*	771	118	0.2	<0.0001	methionine sulfoxide reductase *msrA/msrB*

α Reads/locus after library normalization by DEseq.

β Locus number as annotated in H44/76 WGS (AEQZ00000000).

γ Gene name when annotated in H44/76 WGS (AEQZ00000000).

δ All loci with Benjamini-Hochberg adjusted *p*-value≤0.05.

ε Where available *N. meningitidis* strain MC58 orthologues are noted.

## Discussion

### Positive feedback loop of the σ^E^ operon and control of *msrA/msrB* expression by σ^E^

Previous reports indicated a relatively small σ^E^ operon regulon in pathogenic *Neisseria* when compared to other species with a much wider range of habitats, e.g. *Bacillus subtilis*, where it directs the transcription of many genes that contribute to the formation of mature spores [3], [6], [41]. Here we used RNA-seq to explore this regulon in *N. meningitidis* in greater depth. We found 4 novel genes of which their expression was regulated by σ^E^. We did not detect any similarities between the promoters of these genes and the promoter consensus of the σ^E^ operon and *msrA/msrB* described previously, implicating an indirect effect of σ^E^ [3]. An additional gene, the OpcA superfamily protein NMH_1358, also showed differential expression between H44/76 wt and H44/76Δ*mseR* cells, but this was explained by phase variation. Evidently, the σ^E^ operon and *msrA/msrB* quantitatively show the most dramatic upregulation of sense transcription. The magnitude of differences in expression of the newly identified genes is smaller, which may indicate that the major function of σ^E^ is to provide positive feedback to its own operon and to induce a strong expression of *msrA/msrB*. However, even relatively small transcriptional differences can have a major impact on genome gene expression, e.g. change in transcription factors may propagate among many genes.

### Discovery of novel differentially expressed genes

In general, σ^E^ is found to be involved in the regulation of genes encoding proteins residing in the outer membrane or periplasmic space [2]. Of the 4 newly identified σ^E^ regulated genes, 3 encode outer membrane proteins or secreted proteins. Of these, only one, NMH_0518 (NMB1721), encoding a protein of the PepSY-associated TM helix family of proteins, was upregulated and its protein structure suggests that it is held at the cell wall or is secreted [42]. PepSY domains are present in a diverse family of secreted and cell-wall associated proteins and it has been suggested that they act as protease inhibitors, and as such regulating protease activity in the local environment to the cell, with possible significance for pathogenicity [42]. The 2 other σ^E^ regulated protein coding genes are *nirK (aniA)*, and NMH_2270 (*nspA*); both appeared to be downregulated. NirK is an anaerobically induced outer membrane protein producing nitric oxide through denitrification [43]. Its gene is activated by the fumarate and nitrate reduction regulator FNR when iron concentration is low [44]. When iron concentration is not limiting than *nirK* is repressed by the nitrite-sensitive repressor NsrR, replacing FNR at the *nirK* promoter. NsrR contains a labile Fe-S cluster and when intact it binds to the *nirK* promoter and represses *nirK* [44], [45]. Methionine sulfoxide reductase, ubiquitously present across all organisms and encoded by *msrA/msrB*, might stabilize iron-sulfur clusters as has recently been shown in *Saccharomyces cerevisiae* [4], [46]. We suggest that, considering the pervasive similarities of *msrA/msrB* in both eukaryotes and prokaryotes, high expression of the methionine sulfoxide reductase may favorably affect the stability of the Fe-S complexes of NsrR increasing the relative NsrR-S-Fe concentration shifting the balance towards more repression of *nirK*.

NMH_2270, encoding the Neisserial surface protein NspA is a vaccine candidate that can bind to human factor H and enhances complement resistance [47]. *nspA* is activated by the Ferric Uptake Regulator protein (Fur) when iron concentration is not limiting and downregulated when iron concentrations are low [48]. In conditions with sufficient iron, Fur also downregulates itself via a negative feedback loop. The Fur protein contains Fe-S clusters and in the complexed form it binds to its own promoter and represses transcription of itself. The Fur protein contains Fe-S clusters and again with high expression of methionine sulfoxide reductase the Fe-S clusters in Fur may be stabilized [48]–[50]. Reduced transcription of *fur* was detected in H44/76Δ*mseR* cells albeit not significant, therefore a(n) alternative mechanism(s) may explain the downregulation of *nspA*.

As described by Anders *et al.*, DEseq was designed to improve on previous tools like edgeR, which tends to overestimate differential expression in the lower range of expression, while underestimating differential expression when genes are relatively highly expressed [31], [51], [52]. Nevertheless, examples of the first may still be present, with σ^E^ and *nqrE*, listed in Table 2 as being upregulated in their antisense direction. Both have very low expression in both the H44/76 wt and H44/76Δ*mseR*. Upon closer visual inspection all reads are scattered within these relatively large loci without any apparent organization. They are therefore either near the detection limit of this experiment or not likely to represent any biological relevance. Examples representing the other side of the quantitative transcriptional spectrum are the genes encoding tRNA-Leu; NMH_1084, NMH_1085 and NMH_2191, which show 2.0 to 3.4 fold upregulation (Table S2). Especially, NMH_2191 has a high number of uniquely mapped reads with 224294 readsn the H44/76 wt and 771702 reads in H44/76Δ*mseR* cells. This extremely high amount of reads is seen only with tRNAs, 5S RNA and several mRNAs coding for ribosomal proteins. Increased expression of tRNA-Leu genes can be caused by an increased demand for leucine incorporation in leucine-rich proteins. Analysis of the σ^E^ operon using Composition Profiler reveals relatively high usage of leucine (*p*<0.001) compared to the average usage of this amino acid in the total protein coding content of the H44/76 genome [53]. Therefore, artificially created constitutional overexpression of this operon increases the intracellular demand for leucine dramatically, which provides a biological explanation for the observed increase of reads mapped to three tRNA-Leu loci (NMH_2191, NMH_1084 and NMH_1085).

There are three additional clusters of genes which showed differential expression without reaching significance when adjusted according to Benjamini-Hochberg to control for FDR, comprising 1) a restriction modification system, 2) the leukotoxin type I secretion system and 3) the thiamin biosynthesis operon (Table S2). The increased expression of the restriction modification locus in H44/76Δ*mseR* cells may be caused by phase variation. The methyltransferase (mod) protein NMH1820 (analogous to NMB1375, pseudogene part 1) contains tetrameric AGCC repeats causing a frameshift that leads to a premature stop codon [54]. After a stop codon, ribosomes dissociate from mRNAs, making that part of mRNA less protected against degradation and lowering the number of reads of the mRNA posterior of the stop codon in H44/76 wt [55]. However, Sanger re-sequencing of this repeat region in H44/76 and H44/76Δ*mseR* using genomic DNA extracted from the samples used for RNA-seq did not show differences in the number of tetrameric repeats. Different mechanisms for differential expression, e.g. transcriptional slippage might be involved, but the lack of genomic differences indicate in favor of the current finding of non-significant differential expression [56].

It remains unclear why the leukotoxin type I secretion system is 2-fold downregulated and genes encoding proteins involved in thiamin biosynthesis were 2.2 to 2.8 fold upregulated in H44/76Δ*mseR* cells. Of note, the thiamin biosynthesis pathway also contains enzymes with iron-sulfur clusters which might suggest a relation with the strong upregulation of *msrA*/*msrB* [57]. Interestingly, oxidative stress leads to auxotrophic requirements for thiamin in *Salmonella enterica* and *Erwinia chrysanthemi*. [58], [59]. In *E. chrysanthemi*, thiamin auxothrophy can also results from depletion of the Isc system, which is known to assist Fe-S cluster biogenesis, resulting in drastic alterations in virulence [59]. In meningococci, increased expression of MsrA/MsrB can prevent Fe-S cluster disintegration, thus supporting increased thiamin biosynthesis.

### Differential expression of non-coding RNA transcripts

Only one IGR showed differential expression between H44/76 wt and H44/76Δ*mseR* cells. The expression of a sRNA transcript located in this IGR between NMH_1566 and NMH_1568 is upregulated in the H44/76Δ*mseR* mutant (Figure 3). This σ^E^sRNA contains no open reading frame and a BLAST search shows no similarity on the nucleotide level with species other than *Neisseria*. Putative mRNA targets of this σ^E^sRNA are, in addition to several genes coding for hypothetical proteins, Fur, a NadC transporter family protein and a TetR family transcriptional regulator. By regulating the expression of *fur* it may also effect the transcription of *nspA*. Moreover, regulation of *fur* and the TetR family transcriptional regulator would extend the impact of σ^E^ on the global protein expression pattern. Interaction of many sRNAs and their mRNA targets has been shown to be enhanced by the bacterial sm-like protein Hfq by facilitating base pairing and stabilizing the double stranded RNA complex [60]–[62]. Two proteomic analyses of the influence of deletion of *hfq* on the protein expression patterns of *N. meningitidis* strains MC58 and H44/76 have shown an attenuated phenotype with an extensive profile of differentially expressed proteins [63], [64]. None of the putative mRNA targets of this σ^E^sRNA have been shown to be differentially expressed in these studies. This might imply that the targets identified *in silico* are not relevant *in vivo* or the interaction between σ^E^sRNA and its targets is Hfq independent. Since the sRNA expression is increased in H44/76Δ*mseR* cells one might have anticipated differential expression of its target genes. Nevertheless, none of the putative target genes of the sRNA show differential expression between H44/76 wt and H44/76Δ*mseR* cells. When sRNAs interact with target mRNAs, the latter are often destabilized due to the activity of the RNase E and RNAse III dependent degradosome [55], [65]. As a result, a target mRNA is degraded to smaller fragments which can be observed as a less intense or fragmented signal in northern blots or with a reduced or absent signal in RT-PCRs [62], [66]. RNase III is also used to process total RNA extracted from the bacterial cultures during SOLiD library preparation. In addition, with the SOLiD sequencing protocol employed here, reads between 25 and 50 nts were generated from all RNA molecules with a native or processed size of 50 nts or larger. Together this makes RNA-seq by SOLiD sequencing with the currently used protocol less suitable for detection of sRNA-mRNA interactions. Further study is underway to assess the function of this interesting non-coding sRNA.

### ρ-independent terminator read-through

NMH_2475 and NMH_2476 show increased antisense transcription in H44/76Δ*mseR* cells. This can be explained by read-through past the ρ-independent terminator of the highly expressed *msrA*/*msrB* locus located diametrically oriented just downstream of these genes. This is visualized by small peaks of the blue H44/76Δ*mseR* line on the negative x-axis (representing negative strand expression) at the NMH_2475 and NMH_2476 loci located on the positive strand, while the red H44/76 wt line shows no antisense expression (Figure 1). DEseq analysis shows that sense expression of NMH_2475 and NMH_2476 is not significantly altered; therefore, the potential stereometrical obstruction of opposing RNA polymerases does not seem to influence the expression of these genes. Similar inefficient termination of transcription has been observed in 454 pyrosequencing RNA-seq experiments in *Helicobacter pylori*, confirmed by northern blot probe hybridization with larger RNA molecules than expected [67].

This report provides an in-depth analysis of the concise but complete σ^E^ factor regulon in the Beta-proteobacterium *Neisseria meningitidis*. This relatively small regulon implies a strictly defined response suitable for the relatively stable niche, the human throat, where *N. meningitidis* resides.

## Supporting Information

Table S1 **List of primers used in qRT-PCR experiments.**
(XLS)

Table S2 **List of differentially expressed loci with uncorrected** ***p*** **-values≤0.01.**
(XLS)

Table S3 **Results of qRT-PCR experiments.**
(XLS)

## References

[1] Cases I, de Lorenzo V (2005). Promoters in the environment: transcriptional regulation in its natural context.. Nat Rev Microbiol.

[2] Brooks BE, Buchanan SK (2008). Signaling mechanisms for activation of extracytoplasmic function (ECF) sigma factors.. Biochim Biophys Acta.

[3] Hopman CT, Speijer D, van der Ende A, Pannekoek Y (2010). Identification of a novel anti-sigmaE factor in *Neisseria meningitidis*.. BMC Microbiol.

[4] Ezraty B, Aussel L, Barras F (2005). Methionine sulfoxide reductases in prokaryotes.. Biochim Biophys Acta.

[5] Gruez A, Libiad M, Boschi-Muller S, Branlant G (2010). Structural and biochemical characterization of free methionine-R-sulfoxide reductase from *Neisseria meningitidis*.. J Biol Chem.

[6] Gunesekere IC, Kahler CM, Ryan CS, Snyder LA, Saunders NJ (2006). Ecf, an alternative sigma factor from *Neisseria gonorrhoeae*, controls expression of msrAB, which encodes methionine sulfoxide reductase.. J Bacteriol.

[7] Draghici S, Khatri P, Eklund AC, Szallasi Z (2006). Reliability and reproducibility issues in DNA microarray measurements.. Trends Genet.

[8] Tan PK, Downey TJ, Spitznagel EL, Xu P, Fu D (2003). Evaluation of gene expression measurements from commercial microarray platforms.. Nucleic Acids Res.

[9] Shi L, Reid LH, Jones WD, Shippy R, Warrington JA (2006). The MicroArray Quality Control (MAQC) project shows inter- and intraplatform reproducibility of gene expression measurements.. Nat Biotechnol.

[10] Waters LS, Storz G (2009). Regulatory RNAs in bacteria.. Cell.

[11] Papenfort K, Vogel J (2010). Regulatory RNA in bacterial pathogens.. Cell Host Microbe.

[12] Papenfort K, Pfeiffer V, Mika F, Lucchini S, Hinton JC (2006). SigmaE-dependent small RNAs of Salmonella respond to membrane stress by accelerating global omp mRNA decay.. Mol Microbiol.

[13] Udekwu KI, Wagner EG (2007). Sigma E controls biogenesis of the antisense RNA MicA.. Nucleic Acids Res.

[14] Croucher NJ, Thomson NR (2010). Studying bacterial transcriptomes using RNA-seq.. Curr Opin Microbiol.

[15] Sorek R, Cossart P (2010). Prokaryotic transcriptomics: a new view on regulation, physiology and pathogenicity.. Nat Rev Genet.

[16] Frasch CE, Zollinger WD, Poolman JT (1985). Serotype antigens of *Neisseria meningitidis* and a proposed scheme for designation of serotypes.. Rev Infect Dis.

[17] van der Ende A, Hopman CT, Dankert J (1999). Deletion of porA by recombination between clusters of repetitive extragenic palindromic sequences in *Neisseria meningitidis*.. Infect Immun.

[18] Tettelin H, Saunders NJ, Heidelberg J, Jeffries AC, Nelson KE (2000). Complete genome sequence of *Neisseria meningitidis* serogroup B strain MC58.. Science.

[19] Piet JR, Huis In 't Veld RA, van Schaik BD, van Kampen AH, Baas F (2011). Genome Sequence of *Neisseria meningitidis* Serogroup B Strain H44/76.. J Bacteriol.

[20] Budroni S, Siena E, Hotopp JC, Seib KL, Serruto D (2011). *Neisseria meningitidis* is structured in clades associated with restriction modification systems that modulate homologous recombination.. Proc Natl Acad Sci U S A.

[21] Bhagwat AA, Phadke RP, Wheeler D, Kalantre S, Gudipati M (2003). Computational methods and evaluation of RNA stabilization reagents for genome-wide expression studies.. J Microbiol Methods.

[22] Passalacqua KD, Varadarajan A, Ondov BD, Okou DT, Zwick ME (2009). Structure and complexity of a bacterial transcriptome.. J Bacteriol.

[23] Chomczynski P, Sacchi N (1987). Single-step method of RNA isolation by acid guanidinium thiocyanate-phenol-chloroform extraction.. Anal Biochem.

[24] Schroeder A, Mueller O, Stocker S, Salowsky R, Leiber M (2006). The RIN: an RNA integrity number for assigning integrity values to RNA measurements.. BMC Mol Biol.

[25] Luu-The V, Paquet N, Calvo E, Cumps J (2005). Improved real-time RT-PCR method for high-throughput measurements using second derivative calculation and double correction.. Biotechniques.

[26] Pfaffl MW (2001). A new mathematical model for relative quantification in real-time RT-PCR.. Nucleic Acids Res.

[27] Li H, Durbin R (2009). Fast and accurate short read alignment with Burrows-Wheeler transform.. Bioinformatics.

[28] Li H, Handsaker B, Wysoker A, Fennell T, Ruan J (2009). The Sequence Alignment/Map format and SAMtools.. Bioinformatics.

[29] R Development Core Team (2011). R: A language and environment for statistical computing.

[30] Gentleman RC, Carey VJ, Bates DM, Bolstad B, Dettling M (2004). Bioconductor: open software development for computational biology and bioinformatics.. Genome Biol.

[31] Anders S, Huber W (2010). Differential expression analysis for sequence count data.. Genome Biol.

[32] Benjamini Y, Hochberg Y (1995). Controlling the False Discovery Rate: A Practical and Powerful Approach to Multiple Testing.. J R Statist Soc Ser B.

[33] Reese MG (2001). Application of a time-delay neural network to promoter annotation in the *Drosophila melanogaster* genome.. Comput Chem.

[34] Kingsford CL, Ayanbule K, Salzberg SL (2007). Rapid, accurate, computational discovery of Rho-independent transcription terminators illuminates their relationship to DNA uptake.. Genome Biol.

[35] Zuker M (2003). Mfold web server for nucleic acid folding and hybridization prediction.. Nucleic Acids Res.

[36] Tjaden B (2008). TargetRNA: a tool for predicting targets of small RNA action in bacteria.. Nucleic Acids Res.

[37] Sarkari J, Pandit N, Moxon ER, Achtman M (1994). Variable expression of the Opc outer membrane protein in *Neisseria meningitidis* is caused by size variation of a promoter containing poly-cytidine.. Mol Microbiol.

[38] Moxon R, Bayliss C, Hood D (2006). Bacterial contingency loci: the role of simple sequence DNA repeats in bacterial adaptation.. Annu Rev Genet.

[39] Hegedus Z, Zakrzewska A, Agoston VC, Ordas A, Racz P (2009). Deep sequencing of the zebrafish transcriptome response to mycobacterium infection.. Mol Immunol.

[40] Lavorgna G, Dahary D, Lehner B, Sorek R, Sanderson CM (2004). In search of antisense.. Trends Biochem Sci.

[41] Feucht A, Evans L, Errington J (2003). Identification of sporulation genes by genome-wide analysis of the sigmaE regulon of *Bacillus subtilis*.. Microbiology.

[42] Yeats C, Rawlings ND, Bateman A (2004). The PepSY domain: a regulator of peptidase activity in the microbial environment?. Trends Biochem Sci.

[43] Anjum MF, Stevanin TM, Read RC, Moir JW (2002). Nitric oxide metabolism in *Neisseria meningitidis*.. J Bacteriol.

[44] Rock JD, Thomson MJ, Read RC, Moir JW (2007). Regulation of denitrification genes in *Neisseria meningitidis* by nitric oxide and the repressor NsrR.. J Bacteriol.

[45] Nakano MM, Geng H, Nakano S, Kobayashi K (2006). The nitric oxide-responsive regulator NsrR controls ResDE-dependent gene expression.. J Bacteriol.

[46] Sideri TC, Willetts SA, Avery SV (2009). Methionine sulphoxide reductases protect iron-sulphur clusters from oxidative inactivation in yeast.. Microbiology.

[47] Lewis LA, Ngampasutadol J, Wallace R, Reid JE, Vogel U (2010). The meningococcal vaccine candidate neisserial surface protein A (NspA) binds to factor H and enhances meningococcal resistance to complement.. PLoS Pathog.

[48] Shaik YB, Grogan S, Davey M, Sebastian S, Goswami S (2007). Expression of the iron-activated nspA and secY genes in *Neisseria meningitidis* group B by Fur-dependent and -independent mechanisms.. J Bacteriol.

[49] Delany I, Grifantini R, Bartolini E, Rappuoli R, Scarlato V (2006). Effect of *Neisseria meningitidis* fur mutations on global control of gene transcription.. J Bacteriol.

[50] Grifantini R, Sebastian S, Frigimelica E, Draghi M, Bartolini E (2003). Identification of iron-activated and -repressed Fur-dependent genes by transcriptome analysis of *Neisseria meningitidis* group B.. Proc Natl Acad Sci U S A.

[51] Robinson MD, Smyth GK (2007). Moderated statistical tests for assessing differences in tag abundance.. Bioinformatics.

[52] Robinson MD, McCarthy DJ, Smyth GK (2010). edgeR: a Bioconductor package for differential expression analysis of digital gene expression data.. Bioinformatics.

[53] Vacic V, Uversky VN, Dunker AK, Lonardi S (2007). Composition Profiler: a tool for discovery and visualization of amino acid composition differences.. BMC Bioinformatics.

[54] Srikhanta YN, Fox KL, Jennings MP (2010). The phasevarion: phase variation of type III DNA methyltransferases controls coordinated switching in multiple genes.. Nat Rev Microbiol.

[55] Belasco JG (2010). All things must pass: contrasts and commonalities in eukaryotic and bacterial mRNA decay.. Nat Rev Mol Cell Biol.

[56] Penno C, Hachani A, Biskri L, Sansonetti P, Allaoui A (2006). Transcriptional slippage controls production of type III secretion apparatus components in *Shigella flexneri*.. Mol Microbiol.

[57] Jurgenson CT, Begley TP, Ealick SE (2009). The structural and biochemical foundations of thiamin biosynthesis.. Annu Rev Biochem.

[58] Thorgersen MP, Downs DM (2009). Oxidative stress and disruption of labile iron generate specific auxotrophic requirements in *Salmonella enterica*.. Microbiology.

[59] Rincon-Enriquez G, Crete P, Barras F, Py B (2008). Biogenesis of Fe/S proteins and pathogenicity: IscR plays a key role in allowing *Erwinia chrysanthemi* to adapt to hostile conditions.. Mol Microbiol.

[60] Brennan RG, Link TM (2007). Hfq structure, function and ligand binding.. Curr Opin Microbiol.

[61] Franze de Fernandez MT, Hayward WS, August JT (1972). Bacterial proteins required for replication of phage Qß ribonucleic acid.. J Biol Chem.

[62] Aiba H (2007). Mechanism of RNA silencing by Hfq-binding small RNAs.. Curr Opin Microbiol.

[63] Pannekoek Y, Huis in 't Veld RAG, Hopman CT, Langerak AA, Speijer D (2009). Molecular characterization and identification of proteins regulated by Hfq in *Neisseria meningitidis*.. FEMS Microbiol Lett.

[64] Fantappie L, Metruccio MM, Seib KL, Oriente F, Cartocci E (2009). The RNA chaperone Hfq is involved in stress response and virulence in *Neisseria meningitidis* and is a pleiotropic regulator of protein expression.. Infect Immun.

[65] Viegas SC, Pfeiffer V, Sittka A, Silva IJ, Vogel J (2007). Characterization of the role of ribonucleases in Salmonella small RNA decay.. Nucleic Acids Res.

[66] Massé E, Escorcia FE, Gottesman S (2003). Coupled degradation of a small regulatory RNA and its mRNA targets in *Escherichia coli*.. Genes Dev.

[67] Sharma CM, Hoffmann S, Darfeuille F, Reignier J, Findeiss S (2010). The primary transcriptome of the major human pathogen *Helicobacter pylori*.. Nature.

